# Veterinary Medical Society, Ontario Veterinary College

**Published:** 1898-01

**Authors:** C. W. Fisher

**Affiliations:** Secretary


Secretary.

### VETERINARY MEDICAL SOCIETY, ONTARIO VETERINARY COLLEGE.

AT the meetings of the Society, in connection with the College, the following papers have been read during the past month. Many of the papers and discussions were unusually interesting and instructive.

Essays: "Sheep-rot," by J. A. Raleigh; "Post-partum Hemorrhage," by W. D. Brand; "Hog-cholera and Swine-plague," by B. Royer; "Ringbone," by J. Young; "Castration," by T. Sims; "Tuberculosis and its Relation to the Veterinarian," by C. W. Fisher; "Ergotism," by S. Caldbick; "Veterinary Profession," by J. W. Parks; "Tetanus," by J. S. Pollard; "Lymphangitis," by G. H. Davidson.

Communications: "Pneumonia," by W. A. Campbell; "Simple Ophthalmia in Sea-lion," by J. L. Devereau; "Laceration of Vagina," by W. D. Brand; "Symptomatic Anthrax," by G. T. Elliott; "Bruise of Calcaneo-cuboid Ligament," by J. E. Ellis; "Removal of Carotid and Jugular," by E. R. Stockwell; "Glanders," by T. Lambrechts; "Laminitis," by G. H. Davidson; "Enteritis," by T. J. Fletcher; "Impaction of Colon," by T. Sims; "Mammitis inMare," by J. Young; "Ventral Hernia," by C. H. A. Stevenson; "Parturient Apoplexy," by B. W. Groff; "Lymphangitis," by D. H. McKay; "Eventration," by W. E. Fairbanks.

C. W. FISHER,
Secretary.

